# Complete Genome Sequence of Sporomusa termitida DSM 4440^T^

**DOI:** 10.1128/MRA.00046-20

**Published:** 2020-03-12

**Authors:** Anja Poehlein, Jacqueline Hollensteiner, Annika Dreyer, Ivana Gavrilova, Rolf Daniel

**Affiliations:** a Genomic and Applied Microbiology & Göttingen Genomics Laboratory, Institute of Microbiology and Genetics, Georg-August University of Göttingen, Göttingen, Germany; b Applied Bioinformatics in Microbiology Course of the Molecular Life Sciences: Microbiology, Biotechnology and Biochemistry M.Sc./Ph.D. Program, Georg-August University of Göttingen, Göttingen, Germany; University of Arizona

## Abstract

Sporomusa termitida type strain DSM 4440 was isolated from the intestinal content of the termite Nasutitermes nigriceps. The genome consists of one circular chromosome (5,185,220 bp) and a megaplasmid (131,121 bp) with an overall G+C content of 49.11%. It harbors 4,764 predicted protein-encoding genes.

## ANNOUNCEMENT

The genus *Sporomusa* consists of Gram-negative, spore-forming, and obligate anaerobic bacteria (1). Sporomusa termitida was discovered first in 1988 during the investigation of the function of acetogenic microorganisms in termite species (2). The strain was originally isolated from an enrichment culture derived from gut homogenates of the termite Nasutitermes nigriceps (3). This strain has been chosen for genome sequencing, as it is the type strain of this species.

*S. termitida* DSM 4440 was obtained from the Deutsche Sammlung für Mikroorganismen und Zellkulturen (DSMZ) in Braunschweig, Germany, and delivered as a freeze-dried pellet in a sealed vial. The vial was stored at room temperature. The vial was opened in an anaerobic chamber and directly transferred to the culture medium. The strain was cultivated in DSM medium 311c (DSMZ) containing 0.15 g/liter dl-dithiothreitol and 1.35 g/liter betaine as the substrate under anaerobic conditions at 30°C without shaking. Subsequently, the overnight culture (3 ml) was harvested by centrifugation, and genomic DNA was directly isolated using the MasterPure complete DNA purification kit (Epicentre, Madison, WI, USA). The isolated DNA was stored at –20°C. Illumina sequencing libraries were prepared using the Nextera XT DNA sample preparation kit. Sequencing performed with a MiSeq instrument and reagent kit version 3 (Illumina, San Diego, CA, USA) resulted in 484,653 paired-end reads. For Nanopore sequencing, 1.5 μg high-molecular-weight DNA was used for library preparation, with the ligation sequencing kit 1D (SQK-LSK108) and the native barcode expansion kit (EXP-NBD103; Barcode 3) employed as recommended by the manufacturer (Oxford Nanopore Technologies, Oxford, UK). Sequencing was performed for 48 h using a MinION Mk1B device and a SpotON flow cell R9.4 as recommended by the manufacturer (Oxford Nanopore Technologies). MinKNOW software v15.1.1 was employed for sequencing and Albacore v2.3.1 for demultiplexing. Default parameters were used for all software unless otherwise specified. Sequencing resulted in 56,961 reads with an average length of 2,786 bp (*N*_50_, 18,016 bp). The Illumina reads were quality filtered using Trimmomatic v0.36 (4). Unicycler v0.4.6 was used to perform a hybrid assembly, resulting in a closed circular chromosome (5,185,220 bp) and a megaplasmid (131,121 bp) with an overall G+C content of 49.11%. The obtained coverages were 130-fold (Illumina) and 25-fold (Oxford Nanopore). Annotation with Prokka v1.13.3 (5) revealed the presence of 4,764 predicted protein-encoding genes, 105 tRNA genes, 1 transfer-messenger RNA (tmRNA) gene, and 9 CRISPR regions. Analysis of the *S. termitida* genome with Artemis v17.0.1 revealed a region which encoded the proteins involved in the Wood-Ljungdahl pathway. Genes encoding acetyl-coenzyme A (CoA) synthase (locus tags STER_13690, STER_13720, and STER_13730) were identified. The whole region (locus tags STER_13620 to STER_13960) was homologous to the corresponding region of S. ovata (GenBank accession number ASXP00000000) and S. sphaeroides (LSLJ00000000) (6–8). In addition, putative genes involved in the formation of the Rnf complex (locus tags STER_14250 to STER_14300), which is a proton-transferring protein complex (9), were present in the *S. termitida* genome. Genome analysis using antiSMASH (v5.0.0beta1-f1164c3) (10) revealed five regions important for secondary metabolism, comprising genes encoding bacteriocin synthase, sactipeptide synthase, type III polyketide synthase, and beta-lactone production, as well as gene clusters encoding nonribosomal peptide synthetases.

### Data availability.

This genome sequence has been deposited at DDBJ/ENA/GenBank under the accession numbers CP036259 (chromosome) and CP036260 (plasmid). The corresponding raw sequence data have been deposited at the NCBI SRA database under the accession numbers SRR9822007 and SRR9822008.
